# Development of the Thalassaemia Adult Life Index (ThALI)

**DOI:** 10.1186/s12955-020-01437-6

**Published:** 2020-06-12

**Authors:** Xenya Kantaris, Mark Shevlin, John Porter, Lynn Myers

**Affiliations:** 1 Centre for Public Engagement, Faculty of Health, Social Sciences and Education, Kingston University and St George’s, University of London, London, UK; 2 School of Psychology, Psychology Research Institute, University of Ulster, Londonderry, Co., Londonderry, UK; 3 Department of Haematology, University College London Hospitals (UCLH), London, UK; 4 Formerly of Brunel University, Uxbridge, London, UK

**Keywords:** Beta Thalassaemia Major, development, quality of life

## Abstract

**Background:**

Beta Thalassaemia Major (βTM) is a chronic genetic illness whereby the challenges faced by patients exposes them to increased risk of psychosocial issues. Despite this, a disease-specific tool to measure the impact of this illness on adult patients has yet to be developed.

**Methods:**

In collaboration with βTM adult patients, this study aimed to develop a comprehensive, disease-specific, easy to use psychometrically sound tool to measure the impact of chelation and transfusion dependent βTM in a cross-cultural patient group in England.The Thalassaemia Life Index (ThALI) was developed in two stages – *item generation and pre-testing and item reduction* – in collaboration with service users. Recruited adult patients shaped the design of the instrument including its statements and subscales. Standard item reduction techniques were used to develop the instrument.

**Results:**

The final version of the ThALI encompasses 35 statements and five sub-scales - *general physical health, coping, body image, appearance and confidence, social relationships and autonomy*. This endorses the multidimensionality of quality of life (QoL). The factor structure of the ThALI is highly stable and its internal consistency is high (alpha = 0.87 for the overall scale; 0.83–0.94 for its subscales). The ThALI has sound scaling assumptions, acceptability and score variability. Content validity was confirmed by experts and service user interviewees. The loadings for the items retained were adequate and the item discriminant validity sound.

**Conclusions:**

The ThALI covers the impact of βTM in adult patients. Preliminary testing shows its multidimensionality to be reliable and valid. The national authentication of the tool with patients treated in Centres of Excellence will aim to provide further evidence regarding the ThALI’s psychometric properties. Once authenticated, the ThALI may be utilised in research and in clinical settings to assess the effects of new therapies and/or interventions from the patients’ perspective to inform practice and/or to identify areas of concern.

## Introduction

Beta thalassaemia major (βTM) is a genetic red-cell blood disorder that causes the body to manufacture an abnormal form of haemoglobin which is the protein molecule in red blood cells that carries oxygen from the lungs to the rest of the body. Consequently, red blood cells are destroyed, and severe anaemia develops. The prevalence of βTM is commonplace in people with Mediterranean, Middle Eastern, South Asian, South East Asian and the Far Eastern backgrounds [1]. Globally in 2015, it resulted in 16,800 deaths [2].

Clinical symptoms are broad and variable in severity, usually starting at around six months of age and include growth problems (weak and fragile bones), anaemia (leading to tiredness, weakness and shortness of breath, jaundice), a swollen abdomen caused by an enlarged liver or spleen, and reduced fertility. Treatment for this chronic illness involves regular blood transfusions and chelation therapy. Chelation therapy can be in the form of a subcutaneous infusion which is given via a small pump-like device given up to seven nights per week over 8-12 hours. Alternatively, the chelation treatment can be given in tablet form or a combination of a tablet and the infusion pump [3].

The above stated treatment options have improved the prognosis for patients, but long-term survival remains a challenge. About half of the patients being treated in the UK die before the age of 35 years from failure to adhere to this arduous treatment [4]. Links exist between the burdensome treatment of βTM and increased psychosocial problems [5] which in turn can lead to poor adherence and in some cases death [6]. There is no cure for this chronic, lifelong disease.

Conventionally, objective considerations like changes in health parameters, disease status and costs of care are used to describe disease activity and treatment efficacy in the clinical management and research of βTM patients [7], however, it has become increasingly clear that the perspective of the patient is also a useful and significantly important variable [7]. Metrics underpin clinical research and practice and patient involvement in the content and design of such tools ensures their clinical relevance. It is becoming increasingly customary to evaluate medical/health-related outcomes from the perspective of the patient [8], however, frequently they are not involved in the design and development of instruments even though there is much to learn from them.

Several rigorously developed and psychometrically sound instruments are available for use, but they evaluate the need for psychosocial support and measure the psychosocial effects of new treatments [9] or assess satisfaction and adherence [10]. Such tools are not designed for measuring quality of life (QoL) in βTM adult patients. More specifically, they have not been developed for English-speaking adults (from different origins) diagnosed with chelation and transfusion dependent βTM only [10–12]. Generic standardised and validated QoL questionnaires do exist, like the Medical Outcomes Study Short Form 36 [13] and The WHOQoL-100 [14], however, generic instruments in general do not cover areas of outcome that are highly relevant in specific diseases and are not specific to a disease type [15]. They also have limited responsiveness and/or fail to detect change [16].

In collaboration with βTM adult patients, the aim of this project was to develop a disease-specific instrument to measure the impact of chelation and transfusion dependent βTM in a cross-cultural patient group in England. This article describes the study methodology which includes the involvement and views of patients.

## Methods

### Study setting and participants

In this article, the terms ‘patients’ and ‘service users’ will be used interchangeably. The adult patients involved in this collaboration are National Health Service (NHS) service users.

The study was conducted in collaboration with the patients (adult) recruited. Patient involvement/input was significantly important and ‘carried weight’. The number of service users involved, and the level of their involvement differed at each stage of the project. At both stages of the ThALI’s development, patients were involved in structured activities which will be become apparent as the activity is described in each stage of the development of the ThALI. The study participants encompassed a purposive community and clinical (small) sample of chelation and transfusion dependent adult βTM patients.

### Stage 1 of 2: item generation and pre-testing

The ThALI was developed in accordance with psychometric theory [17] in two stages in collaboration with patients.

### Item generation

In this stage, a preliminary questionnaire and its statements were generated from interviews with 16 patients being treated in NHS Trusts in England. The sample inclusion criteria were that the patients were aged 18 years or above and being treated, managed and cared for in the UK. All patients were undergoing regular reference treatment i.e. chelation and transfusion dependency. The exclusion criterion for the study was difficulty with the command of the English language as the questionnaire was developed in the English (UK) language.

Interviews were conducted at the convenience of the patients at their treatment centre.

Patients were identified and recruited by clinical Red Cell Haematology nurse specialists (CNSs) to represent as much of the diversity of the illness as possible. Patients were identified using Electronic medical records (EMRs) along with the CNSs’ own knowledge of the patients. An interview schedule served as a guide for each interview to ensure consistency of questioning. The interview questions were derived from a comprehensive review of the literature, multidisciplinary expert opinion, (two health research Psychologists, a CNS (Rec Cell Haematology) and two Consultant Haematologists), and the ideas of a small group of patient ambassadors being cared for in one NHS Trust. Questions included, *‘What impact does Thalassaemia have on: family life, friends/social activities/hobbies, work/professional relationships, education, day to day life, body image, future plans’?* After each interview, each interviewee was asked if there was anything more that they wanted to add to the discussion to ensure that all relevant questions/issues were addressed.

The interviews were transcribed verbatim and their content thematically analysed by the two health research Psychologists individually and iteratively; accord on the themes was achieved after discussion amongst these experts. Thematic analysis is an effective and explicit approach for identifying, analysing and reporting themes when research is addressing an exploratory question. It is a flexible yet clearly defined approach that provides an accessible method of analysis to a novice qualitative researcher and supports a realist theoretical approach [18].

### Pre-testing

Patients helped to shape the design of the ThALI, its statements and subscales.

The preliminary questionnaire including its instructions, item-stems, items and response options, was reviewed for content, wording and clinical appropriateness by patients and clinicians [7].

There is no optimum number of participants that is needed for pre-testing an instrument [19] so 10 adult patients with βTM were invited to take part in the pre-testing. This group of patients included 5 patients who were interviewed in stage 1 who were receptive to help with the project after being involved at the interview stage, as well as a small, independent, clinical group of 5 heterogeneous patients with βTM from one of the 5 participating Trusts [7]. Trust clinicians working within the thalassaemia multidisciplinary team were also invited to review the questionnaire individually for clinical appropriateness. This ensured content validity. Their individual reviews were collated by the study researcher.

Patients were asked to complete the questionnaire in the presence of the study investigator and to review it by identifying items, item stems and instructions that were unclear, ambiguous, irrelevant, misleading and/or offensive and to make suggestions for adjustments to the questionnaire [7]. One-to-one, informal discussions using the ‘think aloud’ approach were ‘conducted’ with each patient [7]. This method is used to gather data for the design and development of ‘products’ in psychology and the social sciences and avoids interpretation by the ‘subject’ assuming that simple verbalisation in the discussion process should considered objective data [7, 20].

In this stage, the questionnaire was administered by postal survey to a community sample of 381 adult βTM patients recruited from the UK Thalassaemia charity [7].

### Stage 2 of 2: item reduction

Exploratory Factor Analysis was adopted for item reduction to develop the final version of the ThALI using varimax rotation. The overarching goal of EFA is to identify the underlying relationships/structure between measured variables. It is generally considered to be more of a theory-generating than a theory-testing procedure [17]. Although EFA is not based on theoretical criteria domains of interest/importance can be identified by researchers even though there was no statistical basis and associations may be examined between variables using methods of statistics therefore is suited for EFA as per psychometric theory [21]. This was the case in this research. In using EFA, researchers can identify a sample that exhibits a spread in scores. Exploratory factor analysis (EFA) was used to identify a group of items that appeared to measure the domains of general physical health (GPH), psychological health and personal beliefs – coping (C), psychological health and personal beliefs – body image, appearance and confidence (BIAC), social relationships (SR), and autonomy (A). Although the THALI measure is multidimensional each subscale is intended to be unidimensional. Unidimensional subscales were created using maximum likelihood factor analysis.

Ninety-one patients (male, 47%; female, 53%; age range 19-68; M = 33.74; SD = 9.63) completed the questionnaires at this stage of the study. Table 1 shows more detailed characteristics of this patient sample. The patients involved in the study were representative of the cross-cultural diversity of persons with the illness in question so that the ThALI was sensitive to the needs of this patient group.

**Table 1:** Community sample characteristics

Variable	Value in % or n
**Gender**	
Female	47%
Male	53%
**Age**	
Mean (SD)	33.74 (9.63)
Range	19–68
**Ethnicity**	
Greek Cypriot	36%
Indian	25%
Greek	10%
Middle Eastern	10%
Chinese	8%
White British	5%
Turkish Cypriot	3%
Pakistani	3%
**Educational status**	
No qualifications	7%
G.C.S.E. or equivalent i.e. school leaver qualification(s)	34%
University entry or equivalent qualification(s)	13%
Graduate degree or equivalent qualification(s)	38%
Higher degree or equivalent qualification(s) i.e. postgraduate or PhD	8%
**Employment status**	
Employed	69%
Self-employed	3%
Unemployed	10%
Other (student/housewife)	18%
**Marital status**	
Single	58%
Married	35%
Living together as married	7%
**Dependents**	
Mean (SD)	0.47 (0.99)
(Range)	(0–5)
**Treatment type**	
‘Pump’	32%
Oral chelator drug	34%
Combination	34%
**Illness complication(s)**	
Osteoporosis/Osteoarthritis^a^	28%
Diabetes	22%
Cardiac problems	10%
Hypothyroidism^b^	8%
Impaired glucose tolerance	6%
Asthma	4%
Other (not stated)	4%
Hepatitis C^c^	4%
Insensitivity to pain	4%
Eczema	4%
Coeliac disease^d^	2%
Hormonal Imbalance	2%

^a^Loss of bone tissue causing the bone to become brittle and fracture easily/a common joint disease characterized by degeneration of the cartilage that lines joints or by formation of an outgrowth of bone at the boundary of a joint (osteophyte), leading to pain, stiffness and occasionally loss of function [22]. ^b^The underproduction of thyroid hormones by an underactive thyroid gland [22]. ^c^Infection often transmitted through sharing needles and/or blood transfusions; can progress to cirrhosis (long term damage to liver cells) and/or hepatoma (a type of liver cancer) [22]^d^The lining of the small intestine gets damaged due to hypersentivity to gluten (protein found in wheat, rye and other cereals) [22]

The decision to have 7 items per sub-scale prior to exploratory factor analysis (EFA) was made to optimise consistency and to ensure an appropriate balance between generating a short scale (for quick completion and scoring) and having enough items to generate adequate score variability [7]. The development of scales based on pre-determined number of items to retain has been used frequently to facilitate scale comparisons e.g. General Health Questionnaire-28 (GHQ-28) [7, 23].

Although the ThALI measure aimed to be multidimensional, we hypothesised that the sub-domains/sub-scales would be uni-dimensional. Data was tested using maximum likelihood factor analysis which has much better statistical properties than other methods of estimation in factor analyses such as Principle Component Analysis (PCA) [7, 21].

## Results

### Stage 1 of 2: item generation and pre-testing

#### Item generation

A total of 158 health impact phrases/statements were extracted from the interviews [7]. Statements with a high degree of overlap (commonality and similarity) were rejected. Statements were chosen to avoid idiosyncratic and highly specific responses [7]. Statements regarding positive coping with βTM were excluded since the focus was on the negative impact of βTM on patients’ lives [7]. A 61-item questionnaire was fashioned.

All the phrases/statements, which became the tool’s items, referred to the present and were written correspondingly in the first-person using words deemed familiar to all adults [7]. All the items were written in one direction for clarity and none presented in reverse format [7]. All items were to be answered with the preceding 4 weeks in mind; this timeframe relates to the treatment of this illness i.e. frequency of blood transfusions [7].

Upon examination of the content and wording of the items, 2 distinct question stems were deemed appropriate [7]. Most of the items (n=33) were best represented by the stem, *‘In the past four weeks, I have…* ’[7]. Fifteen items describe general physical symptoms; 18 items describe psychological and personal thinking [7]. Eight items describe body image and are best represented by the stem, *‘In the past four weeks, it has bothered me that…* [7]. The remaining items (n=20) are statements relating to social relationships and autonomy and were best represented by the stem, *‘In the past four weeks…* ’[7]. The most widely-used psychometric response scale for surveys is the 5-point Likert scale [24] and was deemed the most appropriate in this case [7]. Responses are anchored from ‘1’ (not at all), to ‘5’ (extremely) [7]. The higher the score, the poorer the QoL.

Inductive themes, which became the tool’s sub-scales, emerged from the verbatim transcribed interviews of the patients and were captured using key words [7]. The 5 broad emergent themes were: *general physical health (GPH), coping (C), body image, appearance and confidence (BIAC), social relationships (SR) and autonomy (A),* which endorses the multidimensionality of QoL [7, 14]. The interviewees were shown the themes and the organisation of the statements in the questionnaire (Table 2); all were satisfied with the outcome of the analyses. The broad themes encompassed appropriate groups of items. The five sub-scales encompassed the three distinct dimensions of subjective health status, the biopsychosocial impact of illness, in this case of βTM. The experts - the two health research Psychologists, the CNS (REC Cell Haematology) and the two Consultant Haematologists - agreed that the five sub-scales fell naturally into the 3 dimension of health status.

**Table 2:** Themes and Health Impact Statement Organisation

Scale category	Facets incorporated within domains (themes emerged during the extraction process)	Definition of scale category
General physical health (GPH)	- Overall QoL and general health	Self-evaluation of personal physical health status
	- Energy, fatigue/tiredness	
	- Pain and discomfort	
	- Sleep	
Autonomy (A)	- Mobility	Self-evaluation of feelings of dependency and control in usual physical role activities
	- Achievements/normality	
	- Education and work	
	- Activities of daily living	
Coping (C)	- Feelings/emotions	Self-evaluation of psychological upset and/or well-being in relation to purpose drive coping.
	- Thinking and	
	Concentration	
	- Planning ahead	
Body image, appearance and confidence (BIAC)	- Bodily image	Self-evaluation and feelings about physical self
	- Self-esteem and confidence	
Social relationships (SR)	- Relationships and support network	Self-evaluation of limitations in social activities from physical and emotional difficulties.
	- Leisure/social activities	
	- Stigma	

Table 2 shows the facets incorporated within the five themes and the definition of the sub-scale category.

#### Pre-testing

All the opinions of the patients were considered and were very positive. All 10 patients stated that the instructions were easy to follow; five found it easy to complete [7]. Two patients mentioned that the layout was good; two stated that it had good readability and three found it “*straightforward and precise”* [7]. Two patients commented that the questionnaire was “*all-encompassing”* and three stated that the questionnaire was much needed [7]. Seven patients remarked that the content was fine and six found it interesting [7]. All the patients commented that the item *‘I have been angry and frustrated’,* could be separated out into two items and that the word *‘aggressive’* in one of the items could be replaced with the word hostile so not to offend and/or upset patients. Patients did not suggest additional concepts or identify concepts not relevant and/or important to their experience.

The clinicians added one item based upon the physical clinical features of the illness; this was ‘*My arms are long’.*

No items were eliminated. After pre-testing, the preliminary 61 item questionnaire was expanded to 63 items.

#### Data analysis

##### Stage 2 of 2: item reduction

Seven items were chosen per sub-scale with items loading on the factor > 0.40 as per ‘rule of thumb’ [7, 25] to ensure the identification of moderate to very strong relationships. Given the importance of the issues/concerns stated by patients, the authors sought to ensure that the strongest items were identified within a short tool that was fit for purpose. The authors are aware that classes of relationships can be arbitrary. Knowingly they resolved not to identify weak and loose relationships which could have theoretically excluded items that didn’t ‘fit’ into sub-scales. The factor loadings for the items that were retained were all adequate, ranging from 0.66– 0.86 for GPH, 0.80 – 0.88 for C, 0.53 – 0.78 for BIAC, 0.41 – 0.86 for SR and 0.73 – 0.88 for A [7]. Table 3 shows the factors (by sub-scales and items) by size of loadings.

**Table 3:** Order (by size of loadings) in which ThALI statements contribute to Factors (sub-scales)

Factor 1 (GPH)		Factor 2 (C)		Factor 3 (BIAC)		Factor 4 (SR)		Factor 5 (A)	
Item	Loading	Item	Loading	Item	Loading	Item	Loading	Item	Loading
Tired	0.86	Frust.	0.88	Shortened body	0.78	Rejected	0.86	Mobility	0.88
Run down	0.85	Angry	0.84	Short in height	0.77	Penalised	0.79	Lifting	0.87
Energy	0.78	Irritable	0.82	Look younger than peers	0.66	Social life affected	0.79	Walking	0.86
Needed Rest	0.74	Emotional	0.81	Conscious of being different to others	0.66	Unable to form relationships	0.56	Most things difficult (gen)	0.82
Stamina	0.73	Anxious	0.81	Pale complexion	0.58	Limited leisure activities	0.53	Bending	0.82
Aches/pains	0.67	Stressed	0.80	Inferior	0.58	Family life restrictions	0.51	Struggle with life routine	0.77
Dizzy	0.66	Worried	0.80	Self-confidence	0.53	Short with family	0.41	Doing chores	0.73

General physical health (GPH); Coping (C); Body image, appearance and confidence (BIAC); Social relationships (SR); Autonomy (A)

Item discriminant validity was assessed by correlating each item with the total sub-scale across all sub-scales. Within all the sub-scales, most correlations were significant at the 0.01 level (two tailed) [7]. The scores within this sample were widespread. Although the sample size was small, 60% of the correlations were strong (>0.75). As the UK adult thalassaemia population is small [26] this sample size was satisfactory [7, 27].

The descriptive statistics and response data of the 5 sub-scales of the ThALI are reported in Tables 4 and 5. Table 4 essentially shows some descriptive scores for the items within sub-scale. Table 5 shows further competences of the sub-scales.

**Table 4:** THALI descriptive statistics

Psychometric Properties	GPH	C	BIAC	SR	A
Item Mean Score Range	1.46–2.78	1.73–2.38	1.34–1.72	1.31–2.10	0.77–2.20
Item SD Range	2.61–3.22	2.55–3.12	2.15–3.06	2.08–2.65	2.62–5.51
Item Skewness Range	−3.95 – − 2.79	−3.47 – − 3.08	−3.32 – − 2.63	−3.41 – − 2.90	−3.30 – − 1.02
Item correlation with Hypothesised Scale Range	r = 0.72–0.88	r = 0.83–0.91	r = 0.63–0.77	r = 0.62–0.83	r = 0.79–0.90

General physical health (GPH); Coping (C); Body image, appearance and confidence (BIAC); Social relationships (SR); Autonomy (A)

**Table 5:** THALI Acceptability

Psychometric Properties	GPH	C	B	SR	A
Total Possible Score Range per item	7–35	7–35	7–35	7–35	7–35
Total Observed Score Range	9–34	7–34	7–32	7–31	7–35
Mean Observed Score (SD)	19.13	17.45	13.42	13.93	16.14
Floor/Ceiling Effect	No	No	Floor (19.27%)	Floor (15.48%)	No
Skewness	0.39	0.50	0.98	0.88	0.76

General physical health (GPH); Coping (C); Body image, appearance and confidence (BIAC); Social relationships (SR); Autonomy (A)

Each of the sub-scales generated scores between 13.42 and 19.13 out of a possible 35 [7]. The standard deviation of the mean scores per sub-scale are below the sub-scale midpoint (21.00) [7].

The analysis of the internal consistency of the sub-scales was evaluated with Cronbach’s α; all the estimates were high (0.83-0.94; GPH 0.90, C 0.84, BIAC, 0.83, SR 0.83, A 0.94) [7].

Table 6 shows the (Pearson) correlations between the 5 sub-scales. All the correlations were positive and significant seemingly due to the sub-scale measurement of a broader global outcome of QoL [7].

**Table 6:** Subscale Correlation Matrix

	GPH	C	BIAC	SR	A
**GPH**	1.00	0.58(**)	0.36(**)	0.55(**)	0.61(**)
**C**	0.58(**)	1.00	0.49(**)	0.54(**)	0.61(**)
**BIAC**	0.36(**)	0.49(**)	1.00	0.60(**)	0.52(**)
**SR**	0.55(**)	0.54(**)	0.60(**)	1.00	0.78(**)
**A**	0.61(**)	0.61(**)	0.52(**)	0.78(**)	1.00

General physical health (GPH); Coping (C); Body image, appearance and confidence (BIAC); Social relationships (SR); Autonomy (A)

** Correlation is significant at the 0.01 level (2-tailed); *N* = 91 for all correlations

## Discussion

The developed ThALI is a sound disease-specific health outcome measure for the chelation and transfusion dependent βTM adult population. As it has been developed in collaboration with service users, it presents the issues and/or concerns of this patient group. The ThALI supports the 3 distinct dimensions of subjective health in βTM patients and shows that accommodation to this chronic illness is indeed multi-dimensional with dynamically interacting facets [7]. The data are promising; perceived quality of life could possibly play a significant role in predicting adjustment to and/or coping with βTM [7], however, such extrapolations need to await empirical verification. The present study provides an adequate starting point.

Quality of life (QoL) consists of subjective and biological factors that relates to the personal experiences of chronically ill service users [7, 28]. As people with other chronic medical conditions have a higher risk of developing mental health problems [7, 29] it would make sense that healthcare professionals listen to the lived experiences of chronically ill service users to benefit future suffering despite physical health problems. Doctors may be well equipped for the biomedical aspects of care but not for the challenges of understanding the psychological, social, and cultural dimensions of illness and health [30]. This is where the patient/service user voice is needed and why their involvement in the development of a tool to assess the impact of illness on their life is of paramount importance.

### Scoring and interpreting the scores of the ThALI

Completing the ThALI takes approximately 5 minutes. Scores can be obtained for the total scale and/or its subscales. The authors suggest a simplistic preliminary method of interpreting scores. The ThALI categorises scores are 35-69 as limited/few problems, 70-104 as few problems, 105-139 as moderate problems and 140-175 as significant problems [7]. As previously stated, the higher the score, the poorer the QoL.

### Limitations

Whilst the ThALI was indeed developed in collaboration with service users and indeed presents the issues and/or concerns of this patient group the decision to exclude ‘weak’ items in the tool could have resulted in the exclusion of when reducing items from the bigger tool to the final 35 item tool. The ThALI will endeavour to undergo a national authentication with a clinical sample of chelation and transfusion dependent adult patients with βTM who are being cared for at Centres of Excellence in NHS Trusts in England. The ThALI will be administered to this group of service users along with other measures of subjective biopsychosocial functioning. The results will allow further assessment of the reliability and validity of the questionnaire. At this stage, interested adult patients will be recruited and trained by REC Cell Haematology CNSs working at the Centres to administer the questionnaires to their fellow patients. In this way, the implementation of the Therapeutic Engagement Questionnaire (TEQ) [31] within this patient group may be facilitated; the service user researchers can feedback any practicalities in its administration and any patient issues/concerns not already accounted for (if any) within the clinical environments with those with lived experience.

From a statistical viewpoint, correlation coefficients tend to be less reliable when estimated from small samples. However, although the sample size was small 60% of the correlations were strong (> 0.75), all were reliable and the themes few. The UK adult thalassaemia population is small [4] therefore this sample size was sufficient [27]. Studies of this patient group are generally small, volunteer or self-selected groups thus restricting the generalisation of the study findings [32, 33]. In addition, the disadvantage of using the charity database to define the sampling frame in stage 2 is that the ‘psychological’ representativeness of people who join charitable groups is unknown. Further sampling would be essential to estimate the generalisability of the measure for use with people with thalassaemia in general.

With regards to the data analyses at the item reduction stage, we acknowledge that the factor analysis did not control for the different item stems, hence question structure. Although content validity was high, this was not accounted for. Although the ThALI was developed in collaboration with patients more direct involvement by patients could always occur in studies such as this.

### Strengths

The strength of this project derives from the collaboration with service users at both stages of the ThALI’s development. Patients involved in the study represented the diversity of persons with thalassamia so the ThALI is sensitive to the needs of this patient group. The involvement of patients in research enables them to develop a sense of empowerment and provides opportunity to share and allow others to benefit from their unique experience [34].

Patient experience narratives from the interviews in stage 1 led to the development of a metric that is clinically appropriate to those who access health and social care services for their care and treatment. These patients provided a realistic and ‘lived’ representation of living with βTM [22, 35] and sound feedback was given at the pre-testing stage to revise the tool. Patient input contributed considerably to the content and design of the ThALI which determined the development of a ‘patient-friendly’ measure.

## Conclusions

The development of the ThALI in an English adult chelation and transfusion dependent βTM patient population was accomplished in collaboration with patients and is available for use by healthcare professionals and researchers to measure the biopsychosocial impact of βTM. With the aid of service user researchers, further research should aim to use and test the ThALI in other NHS Trusts in England and in countries where prevalence of βTM is commonplace. The THALI was developed for an English-speaking population however due to the ethnic demographic status of βTM patients, the authors support that the tool be translated into other languages.

## Data Availability

All data generated or analysed during this study are included in this published article. For more information about the raw data contact the lead and corresponding author.

## Abbreviations

βTM: Beta thalassaemia major
CNS: Clinical nurse specialist
EMRs: Electronic medical records
GHQ-28: General Health Questionnaire-28
PCA: Principle Component Analysis
TEQ: Therapeutic Engagement Questionnaire
QoL: Quality of life
